# 肺腺癌长链非编码RNA启动子DNA甲基化的生物信息学分析

**DOI:** 10.3779/j.issn.1009-3419.2018.06.08

**Published:** 2018-06-20

**Authors:** 文 张, 少军 李, 楠楠 郭, 英男 赵

**Affiliations:** 100048 北京，中国人民解放军总医院第一附属医院胸外科 First Affiliated Hospital of PLA General Hospital, Beijing 100048, China

**Keywords:** 肺腺癌, LincRNA, DNA甲基化, 预后, Lung adenocarcinoma, LincRNA, DNA methylation, Prognosis

## Abstract

**背景与目的:**

肿瘤相关长链非编码RNA(lincRNA)的异常表达影响癌症的进程, 是肿瘤进展机制研究的热点, 然而其调控机制目前并不清楚。本研究通过生物信息学方法研究肺腺癌lincRNA启动子DNA甲基化水平与基因表达和预后的关系。

**方法:**

从TCGA网站下载肺腺癌Illumina Methylation 450K芯片的全基因组DNA甲基化数据及转录组数据, 分析DNA甲基化在lincRNA基因附近的分布情况及与基因表达的关系; 比较肿瘤组织和癌旁组织DNA甲基化和表达变化。

**结果:**

lincRNA启动子附近DNA甲基化水平较低, 且与基因的表达水平呈负相关。在肿瘤组织和癌旁组织间, 15个lincRNA的DNA甲基化水平存在显著差异, 与其表达呈反向变化, 包括与癌症发生发展相关的*FINDER*基因, 该基因启动子高甲基化的肺腺癌病人预后较差, 与基因表达作用刚好相反。

**结论:**

肺腺癌中一些lincRNA启动子的DNA甲基化水平能调控lincRNA的表达, 进而与肺腺癌的预后有关。

## Abstract (en)

**Background and objective:**

Previous studies have found the lincRNAs play important roles in the occurrence and development of lung adenocarcinoma, but their regulated mechanims are still unclear.The aim of this study is to evaluate the relationship between DNA methylation and lincRNA expression, and the effect of prognosis in lung adenocarcinoma.

**Methods:**

The whole genome Illumina Methylation 450 DNA methylatiuon data and RNAseq for lung adenocarcinoma were download from TCGA.DNA methylation around lincRNA and their relationship to gene expression were analyzed.Their contribution to lung adenocarcinoma were further analyzed by comparing DNA methylation and lincRNA expression in tumor and adjacent tissues.

**Results:**

The methylation level in promoter region was lower than other positions in lincRNA, and was negatively correlated with gene expression.There were 427 lincRNA genes showed differential DNA methylation in their promoter regions in tumor and adjacent tissues.Among these, 15 lincRNA genes showed differential gene expression and negatively correlated with DNA methylation, including *FENDRR* (a tumor progression and metastasis gene) whose high methylation in its promoter showed poor survival in lung adenocarcinoma.

**Conclusion:**

The expression of lincRNA can be regulated by DNA methylation in their promoter regions, and the level of DNA methylation is related to patient prognosis in lung adenocarcinoma.

肺癌是目前对人类健康和生命威胁最严重的肿瘤之一。肺腺癌(lung adenocarcinoma)是肺癌的一种, 属于非小细胞肺癌(non-small cell lung cancer, NSCLC), 在女性及不抽烟者中较为常见^[1, 2]^。lincRNA是一类长度大于200个碱基的具有生物学功能的非编码RNA, 已发现有大量的lincRNA在肿瘤组织中的异常表达以及癌变或者肿瘤的抑制存在一定的联系, 可被用作癌症的生物标志物^[3, 4]^。在肺腺癌中已经发现有一些lincRNA如EINCR1、MALAT1、HOTAIR和P21等的异常表达影响癌症的进程^[5-8]^, 然而其具体的调控机制目前还需进一步研究。

DNA甲基化是染色质修饰的一种, 在不改变DNA序列的情况下, 能改变染色质结构, 进而影响周围基因的表达。DNA甲基化能对原癌基因和抑癌基因的表达进行调控, 在肿瘤发生过程中起十分重要的作用。原癌基因启动子的DNA去甲基化能激活原癌基因的表达, 进而导致癌症的发生。如原癌基因*MYC*的启动子DNA去甲基化与很多癌症的发生发展有关^[9-11]^。而抑癌基因启动子区域DNA甲基化水平的升高会抑制*p53*和*Rb*等抑癌基因的表达, 进而促进了癌症的发生发展^[12, 13]^。

启动子DNA甲基化能调控*lincRNA*基因的表达, 与很多疾病的发生发展有关。在多种肿瘤细胞中发现lincRNA基因*MEG3*的表达显著低于正常组织, 其启动子区域有DNA甲基化现象^[14, 15]^。在肝癌细胞中, MEG3受mir-29a的间接调控。mir-29a能抑制甲基转移酶的活性从而调控MEG3的表达。当用脱氧胞苷或RNA干扰的方法抑制甲基转移酶时, MEG3的表达明显上升^[16]^。另外在结直肠癌细胞系中发现, 一些lincRNA基因在细胞用去甲基化试剂处理后, 表达明显上升^[17]^。肺腺癌中, 系统研究DNA甲基化与lincRNA基因表达的关系及其对癌症的影响相对较少。因此, 本研究利用TCGA网站肺腺癌全基因组DNA甲基化芯片(infinium human methylation 450 beadChip)数据和转录组数据, 分析两者的关系及其在肺腺癌的变化和对预后的影响, 为阐明lincRNA在肺腺癌中的可能调控机制提供参考。

## 1 材料与方法

### 1.1 数据来源

从TCGA网站下载507例基于Illumina全基因组DNA甲基化芯片的肺腺癌DNA甲基化数据及594例肺腺癌RNA-seq转录组数据(2017年5月), 同时下载患者的临床结果数据。

### 1.2 方法

#### 1.2.1 lincRNA基因周围DNA甲基化水平的分析

DNA甲基化水平用芯片每个探针的β值表示。β值越高, 表示甲基化水平越高, β值最大值为1, 最小值为0。从Ensembl下载人类基因组注释数据(GRCh38), 根据lincRNA在基因组上的注释数据, 对于启动子上游2, 000 bp和基因下游2, 000 bp区域, 每100 bp计算癌旁组织中探针的平均DNA甲基化水平。在基因区域, 平均分成20个相同长度区域后, 计算每个区域的平均甲基化水平。

#### 1.2.2 转录组数据处理

下载RNAseq数据后, 基因的表达值为FPKM, 用分位数归一化(quantile normalization)对数据进一步进行处理, 计算癌旁组织每个基因的平均表达水平, 然后按表达值高低把基因平均分成3类, 计算不同表达水平基因的DNA甲基化水平。

#### 1.2.3 DNA甲基化差异分析

对于每个基因转录起始位点上游1, 000 bp区域, 用配对*t*检验的方法计算每个探针在肿瘤组织和癌旁组织间DNA甲基化水平的差异。*P*值用*Bonferroni*进行多重矫正后得到*Q*值同时计算两种组织间平均β值差异。对于每个探针, 如果*Q* < 0.05以及Δβ > 0.1, 则认为该探针在两种组织中DNA甲基化水平有显著差异。如果一个基因上游区域有DNA甲基化差异的探针的甲基化水平都向同一方向变化, 则认为该基因的启动子DNA甲基化水平在两种组织中有显著差异

#### 1.2.4 基因表达差异分析

用配对*t*检验的方法计算每个基因在肿瘤组织和癌旁组织间基因表达水平的变化, *P*值用*Bonferroni*进行多重矫正后得到*Q*值, *Q* < 0.05作为基因显著差异的阈值。

#### 1.2.5 生存分析

对356例既有DNA甲基化数据, 也有临床病理特征及预后信息的标本, 根据*lincRNA*基因启动子区域的DNA甲基化水平, 对肺腺癌患者的预后进行生存分析。总生存时间定义为手术至患者死亡或末次随访的时间。生存分析在R中进行, 采用的工具包为Survival包, 不同DNA甲基化水平患者的生存时间差异用survdiff进行统计。

## 2 结果

### 2.1 *lincRNA*基因DNA甲基化分析

从TCGA下载人类肺腺癌的全基因组450 K DNA甲基化芯片数据, 根据Ensembl的基因注释, 计算癌旁组织*lincRNA*基因周围DNA甲基化的分布情况。与以前的结果类似, 在蛋白基因的基因区域有着较高的DNA甲基化水平, 而离转录起始位点较近的上游区域DNA甲基化水平相对较低(图 1A)。DNA甲基化在*lincRNA*基因的分布情况与蛋白基因相近, 其基因区域的DNA甲基化水平较高, 而转录起始位点附近的启动子区域DNA甲基化水平较低(图 1A)。然而, DNA甲基化在这两种基因上的分布情况存在一定的差异, 在基因区域蛋白基因的DNA甲基化水平显著高于*lincRNA*基因, 而在靠近转录起始位点的启动子区域蛋白基因的DNA甲基化水平低于*lincRNA*基因(图 1A)。

**1:**
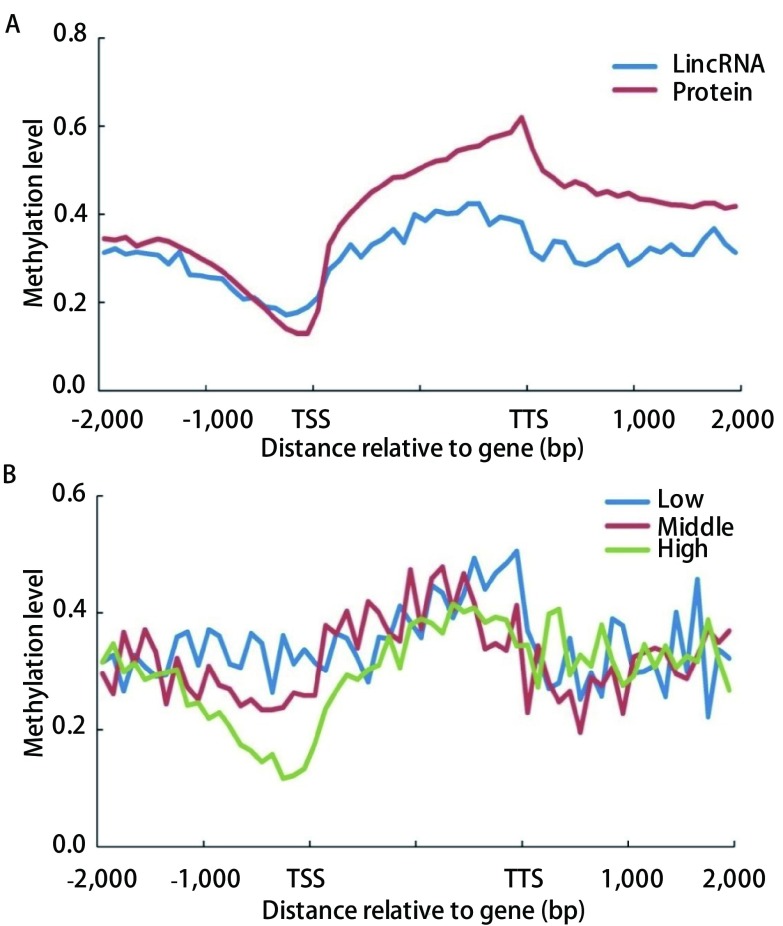
*lincRNA*基因周围DNA甲基化分布分析。A:*lincRNA*基因和蛋白基因周围DNA甲基化分布; B:不同表达水平的*lincRNA*基因DNA甲基化分布。 Distribution of DNA methylation around lincRNA.A:Distribution of DNA methylation around lincRNAs and proteins; B:Distribution of DNA methylation in lincRNAs with different expression levels.

为了研究DNA甲基化对*lincRNA*基因表达的影响, 从TCGA下载507个包括肺腺癌肿瘤组织和癌旁组织的RNA-seq数据, 用分位数归一化后计算肿瘤组织和癌旁组织的*lincRNA*基因平均表达水平, 同时利用癌旁组织计算不同表达水平的*lincRNA*基因DNA甲基化情况。从图 1B可以看出, 不同表达水平的*lincRNA*基因在转录位点上游区域的DNA甲基化存在明显差别, 该处的DNA甲基化对*lincRNA*的基因表达有抑制作用。不同表达水平的*lincRNA*基因区域的甲基化水平没有明显的差异, 表明该位置的DNA甲基化并不影响lincRNA的表达。

### 2.2 肿瘤组织和癌旁组织间lincRNA存在DNA甲基化差异

为了研究lincRNA的DNA甲基化对肺腺癌的影响, 对23例同时含有肿瘤组织和癌旁组织的肺腺癌患者*lincRNA*基因的启动子DNA甲基化进行差异分析。用配对*t*检验的方法计算每个探针在肿瘤组织和癌旁组织间DNA甲基化水平的差异, 共发现420个*lincRNA*基因启动子区域DNA甲基化水平存在显著差异(矫正后的*P* < 0.05, Δβ > 0.1)。其中有280个*lincRNA*基因在肿瘤组织中启动子的DNA甲基化水平明显高于癌旁组织。如图 2所示, 420个基因的启动子区域DNA甲基化热图聚类分析表明, 肿瘤组织和癌旁组织的样本分别聚在不同的分支上, 表明两者的DNA甲基化存在明显差别。同时, 大部分肿瘤组织的DNA甲基化水平明显高于癌旁组织。

**2:**
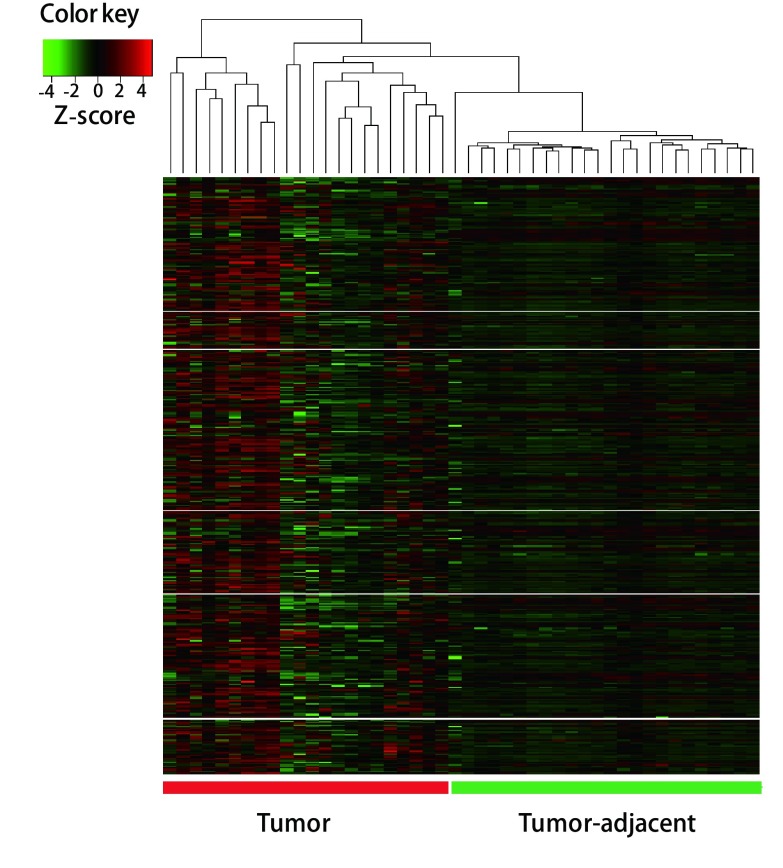
肿瘤组织和癌旁组织*lincRNA*基因启动子DNA甲基化聚类分析 Heatmap analysis of DNA methylation in promoter region of lincRNA in tumor and adjacent tissues

### 2.3 DNA甲基化影响肺腺癌患者的*lincRNA*基因表达

在420个有启动子DNA甲基化变化的lincRNA中, 比较23例同时含有肿瘤组织和癌旁组织肺腺癌病人的lincRNA基因表达变化时发现, 有270个基因的表达变化趋势与甲基化相反。用配对*t*检验的方法计算表明, 在两种组织中, 这270个lincRNA有15个基因的表达有十分显著的差异(Bonferroni多重矫正后结果), 其中有5个*lincRNA*基因的启动子DNA甲基化水平变高, 而其基因表达水平变低, 另外10个*lincRNA*基因的启动子DNA甲基化水平变低, 而其基因表达水平变高(图 3)。表明这15个*lincRNA*基因中启动子DNA甲基化的变化影响基因的表达。

**3:**
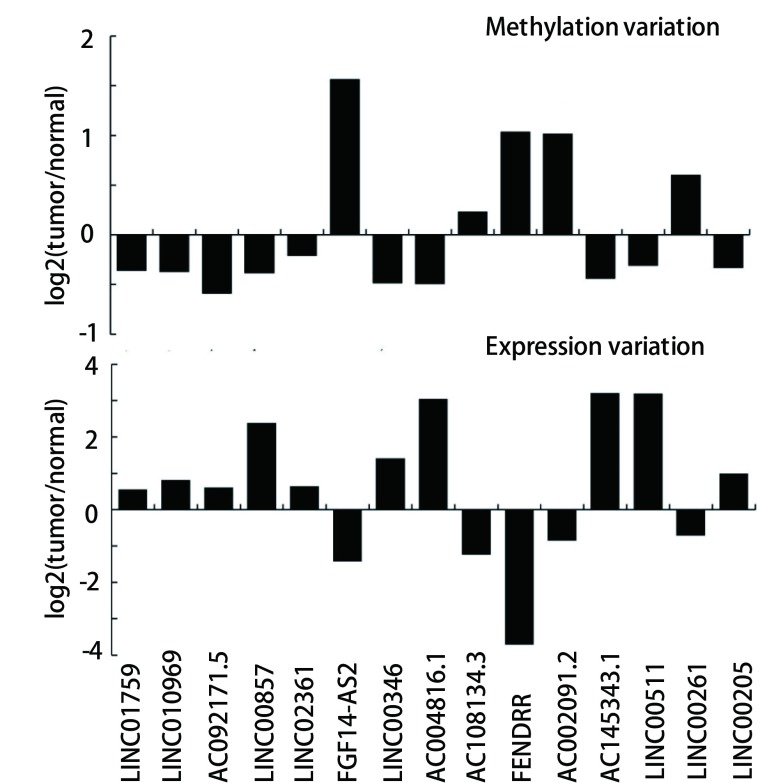
肿瘤组织和癌旁组织*lincRNA*基因启动子DNA甲基化变化与基因表达变化关系 Comparative analysis of DNA methylation in the promoter region of lincRNA and its contribution to gene expression between tumor and adjacent tissues

### 2.4 *lincRNA*基因启动子DNA甲基化水平的变化与肺腺癌患者生存时间有关

在15个启动子有DNA甲基化变化同时基因表达受到影响的lincRNA基因中, FGF14-AS2是一个抑癌基因, 其表达降低与乳腺癌的发生发展有关^[18]^。另一个lincRNA基因FENDRR也是一个抑癌基因, 其表达降低与胃癌的发生发展有关^[19]^。为了进一步研究lincRNA基因启动子DNA甲基化对肺腺癌患者的影响, 对于这15个基因, 按其启动子DNA甲基化水平, 把含有完整预后信息的356个患者分成高甲基化和低甲基化两组, 用R软件中的Survival包计算两者的生存曲线。在这15个*lincRNA*基因中, 有2个基因的DNA甲基化水平与生存时间相关, 为*AC092171.5*和*FENDRR*基因。在这两个基因中, 低甲基化患者相对于高甲基化患者有较长的生存期(图 4A, *P* < 0.05), 该趋势刚好与基因表达相反(图 4B, *P* < 0.05)。

**4:**
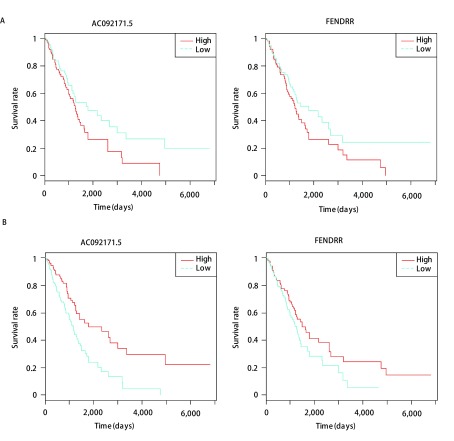
*lincRNA*基因启动子DNA甲基化和基因表达水平与肺腺癌患者的生存时间关系。A:DNA甲基化; B:基因表达。 Survival analysis of patients with different methylation levels in lung adenocarcinoma.A:DNA methylation; B:Gene expression.

## 3 讨论

本研究应用生物信息学手段, 通过分析公用数据库TCGA的人肺腺癌全基因组DNA甲基化数据和RNA-seq数据, 发现*lincRNA*基因启动子区域与基因组区域相比有较低的DNA甲基化现象, 一部分*lincRNA*基因启动子区域的DNA甲基化水平在肿瘤组织和癌旁组织间存在明显差别, 能调控基因的表达。本研究中, 在420个有DNA甲基化水平差异的lincRNA中, 有270个基因的表达变化趋势相反。通过统计检验虽然只有15个变化趋势相反的基因在两种组织中表达值有显著差异, 这可能与我们的统计检验方法较为严格有关。当用较为宽松的*Benjamini-Hochberg*多重矫正方法时, 我们发现130个基因有显著表达差异(矫正后*P* < 0.05)。另外, 一些*lincRNA*基因启动子DNA甲基化水平与患者的预后有关, 包括*FENDRR*和*AC092171.5*。

*FENDRR*基因位于16q24.1, 能结合到PRC2蛋白复合体(polycomb repressive complex 2)上, 调控目标基因的表达^[20]^。在多种肿瘤中发现, FENDRR的表达显著低于正常组织, 且与肿瘤的发生发展有关, 然而其具体的调控机制并不清楚^[19, 21]^。最近有研究^[22]^发现, 在肺癌中, FENDRR能调控肿瘤抑制基因FOXF1的表达, 与肺癌的发生发展有重要联系。通过本文的研究发现, 肿瘤组织中该基因启动子区域的DNA甲基化显著升高, 因而抑制了基因的表达, 从而导致其下游所调控的与癌症发生发展相关的基因表达发生了变化。另外, 该基因启动子DNA甲基化水平越高的患者预后越差, 因此该基因的启动子甲基化可能跟肺腺癌的发生发展以及预后都有关系, 可以作为肺腺癌诊断和预后的一个潜在标志物。AC092171.5启动子DNA甲基化水平在肿瘤组织中显著低于癌旁组织, 然而, 高甲基化患者预后较差, 表明该基因的DNA甲基化与预后也有一定的联系。

越来越多的数据表明DNA甲基化与肺癌的发生发展密切相关。在肺癌患者中已发现一部分蛋白基因的启动子区域存在甲基化现象。*p16*基因是一种重要的抑癌基因, 参与细胞周期蛋白调控。在肿瘤组织中, 该基因启动子区CpG岛甲基化能降低其表达水平, 在肺癌发生发展中起重要作用^[23, 24]^。研究发现, 随着癌症的发展, 肺腺癌中一些基因的DNA甲基化水平也会产生明显的改变。从正常组织到肺腺癌的发展过程中, 这些基因的甲基化程度明显增加^[25]^。因此, 检测DNA甲基化的变化在肺腺癌早期诊断和风险评估有着十分重要的意义。另外, 细胞的高转移特性可能与一些基因的高甲基化相关, DNA甲基化的变化对预测疾病的复发具有一定的意义, 可作为预后效果的一个重要指标^[26]^。

综上, 一些*lincRNA*基因启动子区域的高甲基化会抑制基因的表达, 是其表达的重要调控机制之一。*FENDRR*基因启动子区域的甲基化可以作为肺腺癌诊断及预后的一个重要指标, 该结果同时为进一步研究肺腺癌发病机制提供新的线索。
